# Predicting Sleep and Sleep Stage in Children Using Actigraphy and Heartrate via a Long Short‐Term Memory Deep Learning Algorithm: A Performance Evaluation

**DOI:** 10.1111/jsr.70149

**Published:** 2025-07-17

**Authors:** R. Glenn Weaver, James W. White, Olivia Finnegan, Hongpeng Yang, Zifei Zhong, Keagan Kiely, Catherine Jones, Yan Tong, Srihari Nelakuditi, Rahul Ghosal, David E. Brown, Russ Pate, Gregory J. Welk, Massimiliano de Zambotti, Yuan Wang, Sarah Burkart, Elizabeth L. Adams, Bridget Armstrong, Michael W. Beets

**Affiliations:** ^1^ Department of Exercise Science, Arnold School of Public Health University of South Carolina Columbia South Carolina USA; ^2^ Department of Computer Science and Engineering University of South Carolina Columbia South Carolina USA; ^3^ Kinesiology, College of Health and Human Sciences Iowa State University Ames Iowa USA; ^4^ Center for Health Sciences, SRI Menlo Park California USA

**Keywords:** ambulatory sleep, device agnostic, LSTM, machine learning, youth

## Abstract

Children's ambulatory sleep is commonly measured via actigraphy. However, traditional actigraphy measured sleep (e.g., Sadeh algorithm) struggles to predict wake (i.e., specificity, values typically < 70) and cannot predict sleep stages. Long short‐term memory (LSTM) is a machine learning algorithm that may address these deficiencies. This study evaluated the agreement of LSTM sleep estimates from actigraphy and heartrate (HR) data with polysomnography (PSG). Children (*N* = 238, 5–12 years, 52.8% male, 50% Black 31.9% White) participated in an overnight laboratory polysomnography. Participants were referred because of suspected sleep disruptions. Children wore an ActiGraph GT9X accelerometer and two of three consumer wearables (i.e., Apple Watch Series 7, Fitbit Sense, Garmin Vivoactive 4) on their non‐dominant wrist during the polysomnogram. LSTM estimated sleep versus wake and sleep stage (wake, not‐REM, REM) using raw actigraphy and HR data for each 30‐s epoch. Logistic regression and random forest were also estimated as a benchmark for performance with which to compare the LSTM results. A 10‐fold cross‐validation technique was employed, and confusion matrices were constructed. Sensitivity and specificity were calculated to assess the agreement between research‐grade and consumer wearables with the criterion polysomnography. For sleep versus wake classification, LSTM outperformed logistic regression and random forest with accuracy ranging from 94.1 to 95.1, sensitivity ranging from 94.9 to 95.9 across different devices, and specificity ranging from 84.5 to 89.6. The addition of HR improved the prediction of sleep stages but not binary sleep versus wake. LSTM is promising for predicting sleep and sleep staging from actigraphy data, and HR may improve sleep stage prediction.

## 1 Introduction

Measuring ambulatory sleep accurately is essential for understanding its relationship with children's health, and there is growing evidence that sleep architecture (i.e., light, deep, and rapid‐eye‐movement [REM] sleep) in addition to sleep duration and timing is critical for a deeper understanding of sleep's relationship with children's health (Arana‐Lechuga et al. 2008; Palmer and Alfano 2017; Vendrame et al. 2008). The most common method for measuring ambulatory sleep is wrist‐placed actigraphy (Galland et al. 2018; Goldstone et al. 2018). However, wrist‐placed actigraphy is limited in at least two critical ways. First, the methods used to distill wrist‐placed actigraphy have chronically produced estimates with low specificity (e.g., specificity of ≤ 70) (Goldstone et al. 2018), indicating that these methods struggle to predict periods of wake in the actigraphy data throughout the night. As a result, they tend to overestimate sleep compared to gold‐standard polysomnography (PSG). The second limitation is that the methods used to process wrist‐placed actigraphy are unable to discern sleep stage accurately (Goldstone et al. 2018; Van De Water et al. 2011; de Zambotti et al. 2018; de Zambotti et al. 2016; Yuan et al. 2024). Previous work has demonstrated that the addition of heartrate (HR) to actigraphy improves the prediction of sleep stage (Lujan et al. 2021; Aktaruzzaman et al. 2017) and may improve specificity estimates. However, it is rare that HR is incorporated into research‐grade actigraphy devices because adding the HR sensor reduces the run time of wearable monitors from weeks to days, due to increased power demand (Goldstone et al. 2018). Research‐grade devices that include an HR sensor are also exceptionally expensive, ranging from $600 to $2000 per device (Empatica 2023; Actigraph 2023). An alternative approach is to use consumer wearables that incorporate actigraphy and HR and are often cheaper than research‐grade devices. However, the proprietary nature of the sleep estimates produced from the algorithms employed in consumer wearables limits their utility in clinical and research settings because manufacturers could make substantial estimate‐altering changes to these algorithms at any time without alerting the public (Goldstone et al. 2018; De Zambotti et al. 2019). For these reasons, better methods are needed for distilling actigraphy data into sleep and sleep stage.

One strategy for addressing the limitations of actigraphy is to explore alternative analysis strategies. One particularly promising technique is long short‐term memory (LSTM) (Hochreiter 1997). LSTM is a deep learning method and may be ideal for sleep classification because deep learning methods are efficient at handling large amounts of data that are typical with sleep measurement and they can identify relevant features automatically from raw data, which obviates the burden of manually identifying relevant features. Further, LSTM is designed by assuming that data has dependencies over time (i.e., the current prediction depends on what occurred previously and what follows). Thus, it capitalises on these dependencies to predict an outcome. This is particularly applicable in the context of sleep, because sleep is phasic (i.e., composed of different phases) and ordered (i.e., N1 comes before N2 and N3 comes before REM). The primary application of LSTM has been to automatically predict binary sleep versus wake and sleep stages from data streams commonly included in PSG (e.g., electroencephalography, electrocardiogram, electrooculography) (Michielli et al. 2019; Phan et al. 2019; Radha et al. 2019), eliminating the need for manual scoring of PSG data that is time‐consuming and therefore expensive. LSTM has only recently been applied to wrist‐placed actigraphy data (Sano et al. 2018; Wulterkens et al. 2021; Olsen et al. 2022; Palotti et al. 2019; Haghayegh et al. 2020; Sathyanarayana et al. 2016). While this work shows promise, these studies are limited in key ways. For instance, these studies are primarily focused on adults, with one study including a small sample of children (*n* = 48) (Wulterkens et al. 2021), and one study including a sample of adolescents (Sathyanarayana et al. 2016). Only three studies have explored LSTM's ability to classify sleep using actigraphy exclusively (Sano et al. 2018; Palotti et al. 2019; Sathyanarayana et al. 2016). However, two of these studies are exclusively in adults and only one of them used the gold standard ground truth, polysomnography, to predict sleep (Palotti et al. 2019). Further, one study focused on sleep efficiency exclusively as an outcome (Sathyanarayana et al. 2016).

In this paper, we build on existing literature by applying an LSTM framework in a novel way—specifically, using only acceleration data to classify sleep, which enhances applicability outside of laboratory settings. Additionally, we explore whether incorporating HR data improves classification accuracy. Logistic regression (LR) and random forest (RF) algorithms were used to establish a performance baseline for comparison with LSTM.

## 2 Methods

### 2.1 Setting and Participants

Participants were recruited from a sleep clinic located at a local children's hospital in the greater metropolitan area of a midsized southeastern city in the United States. Children at the clinic were referred by their paediatrician for an overnight sleep study because of disrupted sleep due to snoring, enlarged tonsils, or restless sleep. Parents and children were presented with the opportunity to participate in the current study after intake at the paediatric sleep clinic. Interested parents then completed an informed consent document, and their child provided verbal assent. Children were excluded from participation if they were younger than five or older than 12 years. Families received a $40 gift card for participation upon completion of the study. Prior to the enrolment of the first participant, study procedures were approved by the first author's institutional review board.

### 2.2 Sample Size

The sample size was selected based on best practice guidelines. Guidelines call for sufficient numbers of participants (i.e., 10/group) to represent different ages and relevant confounders (Kim et al. 2012; Freedson et al. 2005; Freedson and Miller 2000). For our study, the age range was 5–12 years, which we divided into 3 age categories (5–7, 8–10, 11–12 years). The confounders we considered were sex and obstructive sleep apnea diagnosis (obstructive sleep apnea vs. no obstructive sleep apnea as determined by clinical evaluation by a sleep specialist and consistent with the American Academy of Sleep Medicine). Thus, to get at least 10 participants per group, we recruited enough children so that ~120 children wore each wrist‐placed device (research grade and consumer wearables).

### 2.3 Procedures

Data were collected from March 2022 to June 2024. Trained sleep technicians performed an overnight PSG on participants in the sleep clinic. Children wore the wrist‐placed devices on their non‐dominant wrist simultaneously with the PSG. All devices were placed on the child by a sleep technician and removed at the conclusion of the PSG. After placement of devices, children self‐selected bedtime and wakeup time. A total of 240 children participated in the study, with 238 children included in the final analytic sample because they had data from PSG and all three accelerometer devices concordantly. Two children were excluded from the analysis because of incomplete PSG data.

### 2.4 Instrumentation

*Polysomnography*. Trained sleep technicians performed an overnight PSG on participants in the sleep clinic using the Nihon Kohden PSG‐1100 system with Polysmith software (Tokyo, Japan). Two electrooculograms, two leg electromyographies (EMG), 3‐lead electrocardiograms, chin EMG, electroencephalogram, oxygen saturation, snoring microphone, abdominal respiratory movement, oronasal flow thermistor, nasal pressure transducer, chest respiratory movement, plethysmography, and end‐tidal CO_2_ were included in the monitored channels. A paediatric sleep specialist scored the data in 30‐s epochs according to the American Academy of Sleep Medicine paediatric scoring rules (Berry et al. 2017; Berry et al. 2012).

*Research Grade and Consumer Wearable Actigraphy and HR*. Children wore three accelerometer devices on their non‐dominant wrist simultaneously with the PSG. The devices included a research grade ActiGraph GT9X Link (ActiGraph; ActiGraph LLC, Pensacola, FL), and two of three consumer wearable devices: an Apple Watch Series 7 (Apple; Apple Technology Company, Cupertino, CA), a Garmin Vivoactive 4 (Garmin; Garmin Ltd., Olathe, KS), and/or Fitbit Sense (Fitbit; Google LLC, San Francisco, CA). All devices were placed on the child by a sleep technician and removed at the conclusion of the PSG. Actigraphy data was extracted from the research grade device via the Actilife software (ActiGraph LLC, Pensacola, FL). The raw signal from the electrocardiogram was processed through Kubios HRV Scientific (version 4.0.2, Kuopio, Finland) to extract estimates from HR beats per minute (Tarvainen et al. 2014). Kubios was set to a medium automatic signal quality detection setting, with a 5% acceptance threshold, a 4 Hz interpolation rate, and a smoothing parameter of 500 (Tarvainen et al. 2014). The actigraphy and HR data was extracted from the consumer wearable devices via user‐written apps that leverage the device‐specific API to collect the underlying sensor data on the respective devices. RawLogger (version 1.0.20211201a) was used for the Garmin device, while SensorLog (version 5.2) was used for the Apple device. RawLogger is available for download through the Connect IQTM store on the Garmin ConnectTM app, and SensorLog is available for download through the iOS App Store. For Fitbit, the research team developed a custom application (Slogger). This application leveraged the Fitbit application programming interface to record and export the raw actigraphy data collected via Fitbit. The code for the custom Fitbit app is available at https://github.com/ACOI‐UofSC/Slog_HR. Sampling frequencies from 25 to 100 Hz were recorded based on the capabilities of the ActiGraph (100 Hz), Apple (50 Hz), Garmin (25 Hz), and Fitbit (50 Hz). During the study period, the Apple Watch started on firmware version 8.5 and had 5 firmware updates (8.5.1, 8.6, 8.7, 9, and 9.0.2). The Garmin Vivoactive 4 started on firmware version 6.8 and had 5 firmware updates (6.84, 6.9, 7.01, and 7.2, 7.3) and the Fitbit Sense started on firmware version 128.6.16 and had 1 firmware update (128.6.17). The ActiGraph GT9X Link was on firmware version 1.7.2 and did not update during the study.

### 2.5 Data Processing

In this study, the features used for prediction in the machine learning models were calculated from raw motion and HR data. The motion data included three‐axis accelerometer readings (x, y, and z) and their magnitude, while the HR data provided temporal trends in cardiovascular activity. These raw signals were processed to extract meaningful features for each window using the packages GGIR (Migueles et al. 2019), Two‐Level behaviour classification (TLBC) (Ellis et al. 2016), and Biobank (Doherty et al. 2017). These sources provided a robust set of statistical and summary features such as the mean, standard deviation, range, and activity counts over fixed time windows. HR was included in beats per minute. In total, 145 features were extracted, and all features are listed in Supplementary Table 1.

### 2.6 Feature Selection

Feature selection was tailored to leverage the specific strengths of each model (i.e., RF, LR, and LSTM) by using well‐established features for RF and LR from prior research, while selecting frequency‐domain features for LSTM to align with its sequential processing capabilities.

For RF and LR, feature selection utilised all 145 features. The LSTM model, by contrast, was designed to use frequency‐domain information, which is particularly useful for predicting cyclic sleep–wake behaviours. For LSTM, the input features consisted of frequency components derived from the motion data's x, y, z, magnitude, and HR signals. Frequency‐domain features were extracted by segmenting the raw data into 30‐s non‐overlapping epochs. Within each epoch, spectral features were computed using the TLBC framework, which internally applies a short‐time Fourier transform to overlapping 1‐s windows and averages the resulting power spectra across time. For logistic regression and random forest models, we used the first 15 FFT components (FFT0 to FFT14) as generated by TLBC. For the LSTM model, we extended the number of components to 30 (FFT0 to FFT29) to provide a broader spectral representation suitable for capturing long‐range temporal dependencies.

### 2.7 Outcomes

Data were handled in two ways. First, in the binary approach, sleep stage data from the PSG were flattened into wake and sleep only. In the second sleep stage approach, data from the PSG were grouped into wake, light sleep (stages N1‐N2), deep sleep (stage N3), and REM sleep. Models were then created to predict binary or sleep stage for each algorithm and dataset.

### 2.8 Model Training

In machine learning, there are two important phases in developing and evaluating the model. First, there is the Training Phase, where the model is fed the training dataset, which includes the selected features. In this phase, the model learns how to best use the features to predict the desired outcome. Then, there is the Testing Phase when model performance is evaluated with a separate testing dataset. All machine learning models were evaluated using 10‐fold cross‐validation. The model's performance was averaged across all folds to estimate its accuracy and generalizability. This method helps ensure that the model performs well across different subsets of data, reducing the likelihood of overfitting. In our model training process, we employed a weighted loss function to address class imbalances in the dataset. Class weights were computed using balanced weighting scheme based on class frequency (King and Zeng 2001). Specifically, the weight assigned to each class was computed as $w_{c}=\frac{n_{\text{samples}}}{n_{\text{classes}}\times n_{c}}$, where $n_{\text{samples}}$ is the total number of training samples, $n_{\text{classes}}$ is the number of classes and $n_{c}$ is the number of samples in class c. This method assigns greater weight to underrepresented classes and lower weight to majority classes, ensuring that all classes contribute equally to the learning process. The resulting weights were incorporated into the loss function used for model optimization. By assigning different weights to each class, the model is guided to pay more attention to underrepresented classes, thereby improving overall performance across all classes.

In order to facilitate comparisons between devices, training was completed on three separate subsets of data. For the research grade devices, there were three subsets of data. The first subset included research grade device data when participants were wearing an Apple device, the second subset included research grade device data when participants were wearing a Fitbit device, and the third subset included research grade device data when participants were wearing a Garmin device. There was also Apple device data, Fitbit device data, and Garmin device data. This led to six distinct datasets: (1) research grade data with Apple participants, (2) research grade data with Fitbit participants, (3) research grade data with Garmin participants, (4) Apple data, (5) Fitbit data, and (6) Garmin data. Model training for each algorithm was completed on each dataset separately. The algorithms included in the current study were LR, RF, and LSTM. LR is a classic machine learning technique that allows the analysis of dichotomous outcomes like sleep/wake (LaValley 2008). RF is a popular supervised machine learning algorithm that fits several decision tree classifiers on sub‐samples of the dataset and averages to optimise predictive accuracy. This functionality helps to reduce the threat of overfitting (Breiman 2001). LSTM, a variant of recurrent neural networks (RNNs), is designed to effectively capture long‐term dependencies within sequential data by using memory cells that can retain relevant information over extended periods. In our study, we designed a nested Local–Global LSTM that consists of two primary LSTM components: a local LSTM layer and a global LSTM layer. The local LSTM layer is designed to extract features within each time window, capturing dependencies in the frequency components. The global LSTM layer models these time‐window‐specific features across the entire time sequence, incorporating a broader context to capture long‐term dependencies. This approach allows the model to recognise both localised frequency patterns and overarching temporal structures, enhancing its ability to predict sleep–wake cycles by identifying distinctive frequency characteristics associated with periods of sleep and wakefulness. The final output layer uses this integrated information to predict class probabilities, leveraging both short‐ and long‐term frequency patterns in the data. In addition, our Local–Global LSTM model operates differently by working with frequency‐domain data derived from motion signals, rather than directly using raw time‐series data. Motion data collected over a specific period is transformed into the frequency domain, and the resulting frequency components are treated as sequential steps for the Local LSTM. Code for the final LSTM models can be found here: https://github.com/ACOI‐UofSC/LSTM‐Sleep.

### 2.9 Machine Learning Model Hyperparameters

Hyperparameters in machine learning are key configuration variables, such as the number of nodes and layers, that control model training and directly influence model performance. For LR and RF, grid search was used with 3‐fold cross‐validation on the training dataset to optimise these hyperparameters. The search ranges were as follows: for LR, the complexity parameter $c=\left[10^{-3},10^{-2},\ldots ,10^{2},10^{3}\right]$ was varied across, with the penalty set to either ‘l1’ or ‘l2’. For RF, the maximum tree depth was set to 10, 50, or 100. For the LSTM model, we use a bidirectional LSTM, and the hidden state size was evaluated over (64, 128, and 256), the learning rate over (0.0001, 0.001, and 0.01), and the dropout rate over (0.0, 0.1, and 0.3). The final selected configuration, hidden state size of 128, learning rate of 0.0001, and dropout of 0.1, was chosen based on performance. These parameters were tuned to optimise the model's ability to identify frequency‐based patterns within sequential data, supporting accurate classification across sleep and wake states.

### 2.10 Evaluation Metrics

Consistent with best practice (Menghini et al. 2020; de Zambotti et al. 2022), data were temporally synchronised at the epoch level and confined between lights‐off and lights‐on. All analyses were conducted in accordance with the recommendations for “Rigorous performance evaluation (previously, “validation”*) for informed use of new technologies for sleep health measurement”* of de Zambotti and colleagues (de Zambotti et al. 2022). Analyses were completed with the accompanying open‐source R code found here: https://sri‐human‐sleep.github.io/sleep‐trackers‐performance/AnalyticalPipeline_v1.0.0.html.

Consistent with the standardised framework, discrepancy and epoch‐by‐epoch analyses were completed for the raw actigraphy and HR data collected from each device (i.e., ActiGraph and ECG, Apple, Fitbit, and Garmin) compared to the PSG findings. For the discrepancy analysis, outcomes of interest included total sleep time (TST‐minutes, calculated as the sum of epochs classified as sleep × epoch length/60), sleep efficiency (SE‐percentage, calculated as the percentage of TST/time in bed), SOL (minutes, calculated as the sum of epochs classified as wake before the first sleep epoch × epoch length/60), wake after sleep onset (WASO‐minutes, calculated as the sum of epochs classified as wake after the first sleep epoch × epoch length/60), light sleep (minutes, calculated as the sum of epochs classified as light sleep × epoch length/60), deep sleep (minutes, calculated as the sum of epochs classified as deep sleep × epoch length/60), and REM sleep (minutes, calculated as the sum of epochs classified as REM sleep × epoch length/60). Discrepancy analysis was calculated at the individual and group levels. Individual discrepancy analysis included calculating the mean absolute error (MAE) between TST, SE, SOL, WASO, light sleep, deep sleep, and REM that were estimated via our machine learning models and measured via PSG. Subsequent group level discrepancies were calculated using the MAE computed at the individual level to account for and estimate the systematic and the random component of measurement error in the assessed device. Bland–Altman plots were also constructed for the strongest performing model only (Krouwer 2008). Bland–Altman plots present the systematic bias as well as 95% limits of agreement (LOA) for our best‐performing algorithm (LSTM). The calculation of the systematic bias and LOAs is contingent upon at least three assumptions: (1) bias should be independent of measurement size, (2) homoscedasticity (error should be uniform across measurement size), and (3) bias should be normally distributed. The R code implemented here tests these assumptions and then computes the LOAs accordingly. Details of how the assumptions were tested and the LOAs were subsequently calculated are presented elsewhere (Menghini et al. 2020).

Following the discrepancy analysis, individual and group epoch‐by‐epoch analyses were conducted. For the individual epoch‐by‐epoch analysis, accuracy, sensitivity, and specificity were calculated for each participant's data. Accuracy was defined as the proportion of correctly classified epochs, where correctly classified = agrees with PSG. Sensitivity was defined as the proportion of correctly classified sleep epochs, and specificity was defined as the proportion of correctly classified wake epochs. Again, group‐level epoch‐by‐epoch analyses were then calculated using the accuracy, sensitivity, and specificity estimates computed at the individual level to account for and estimate the systematic and random component of measurement error in the assessed device/s. Details of this process are presented elsewhere (Menghini et al. 2020).

## 3 Results

Demographic characteristics are presented in Table 1. A total of 141, 116, and 136 children wore the Apple, Fitbit, and Garmin devices, respectively. In terms of race, the majority of participants were Black (i.e., 50%) with a mean BMI z‐score of 1.2, which was indicative of the fact that most participants were overweight (i.e., 11.3%) or obese (i.e., 45.8%). Most children were diagnosed with OSA, but the majority of those diagnosed were categorised as mild. Binary sleep and sleep stage estimates produced by all devices and algorithms are presented in Table 2.

**TABLE 1:** Analytic sample demographics.

Number of participants	238	
ActiGraph + ECG (%)	238	(100.0)
Apple (%)	141	(59.2)
Fitbit (%)	116	(48.7)
Garmin (%)	136	(57.1)
Male (%)	124	(52.8)
Mean Age (SD)	8.6	(2.2)
Race		
White non‐Hispanic (%)	76	(31.9)
Black (%)	119	(50.0)
Other Race (%)	43	(18.1)
(*including all races other than Black or White*)		
Mean BMI (SD)	22.4	(8.1)
Mean BMI z‐score (SD)	1.2	(1.4)
Mean BMI Percentile (SD)	75.2	(30.8)
Weight Status		
Underweight (%)	8	(3.4)
Normal Weight (%)	94	(39.5)
Overweight (%)	27	(11.3)
Obese (%)	109	(45.8)
Sleep Diagnosis		
None	42	(17.7)
Mild Obstructive Sleep Apnea	112	(47.1)
Moderate Obstructive Sleep Apnea	38	(16.0)
Severe Obstructive Sleep Apnea	46	(19.3)

**TABLE 2:** Binary sleep and sleep stage estimates produced by all devices and algorithms.

Algorithm	Dataset	Device	
				Binary (sleep/wake)							
TST	(SD)	SE	(SD)	SOL	(SD)	WASO	(SD)
LR	Apple	Polysomnography		321.4	(81.1)	85.2	(11.0)	25.0	(29.1)	31.2	(29.3)
		Research Grade	no HR	295.4	(77.9)	78.1	(11.2)	11.0	(16.7)	71.2	(44.0)
			HR	295.4	(77.0)	78.1	(10.6)	11.8	(16.6)	70.4	(42.9)
		Apple	no HR	301.2	(72.6)	76.8	(11.9)	14.7	(23.1)	76.0	(46.9)
			HR	301.1	(71.9)	76.8	(11.4)	16.8	(24.9)	74.0	(44.3)
	Fitbit	Polysomnography		341.2	(82.3)	85.4	(12.6)	27.8	(30.0)	28.8	(25.1)
		Research grade	no HR	305.1	(79.0)	79.2	(10.4)	10.9	(15.8)	68.3	(38.6)
			HR	304.5	(78.1)	79.0	(10.0)	11.2	(15.8)	68.6	(37.5)
		Fitbit	no HR	312.5	(75.4)	78.1	(11.0)	12.2	(18.8)	73.1	(33.0)
			HR	312.1	(75.6)	77.9	(10.7)	14.1	(21.3)	71.6	(30.2)
	Garmin	Polysomnography		350.7	(65.7)	84.3	(10.7)	29.3	(29.9)	35.2	(28.0)
		Research Grade	no HR	293.3	(79.5)	77.9	(10.7)	9.7	(15.5)	71.7	(41.3)
			HR	292.2	(79.0)	77.6	(10.4)	11.1	(15.7)	71.4	(40.2)
		Garmin	no HR	322.6	(61.6)	77.8	(11.4)	12.0	(18.1)	80.6	(45.0)
			HR	321.6	(61.5)	77.5	(11.2)	13.1	(19.1)	80.5	(43.7)
RF	Apple	Polysomnography		321.4	(81.1)	85.2	(11.0)	25.0	(29.1)	31.2	(29.3)
		Research Grade	no HR	302.0	(79.1)	79.8	(11.2)	10.2	(16.5)	65.5	(44.0)
			HR	303.6	(78.9)	80.2	(10.7)	11.0	(17.5)	63.0	(42.0)
		Apple	no HR	307.0	(73.8)	78.2	(12.0)	14.7	(22.7)	70.2	(45.4)
			HR	307.9	(73.5)	78.4	(11.7)	17.3	(25.9)	66.6	(43.6)
	Fitbit	Polysomnography		341.2	(82.3)	85.4	(12.6)	27.8	(30.0)	28.8	(25.1)
		Research grade	no HR	313.6	(80.3)	81.3	(9.7)	9.8	(15.4)	60.9	(34.1)
			HR	316.2	(79.1)	82.0	(9.0)	10.4	(16.4)	57.6	(31.6)
		Fitbit	no HR	318.3	(76.3)	79.7	(11.0)	14.4	(23.4)	65.1	(30.3)
			HR	319.2	(75.9)	79.9	(10.9)	15.2	(23.4)	63.4	(29.3)
	Garmin	Polysomnography		350.7	(65.7)	84.3	(10.7)	29.3	(29.9)	35.2	(28.0)
		Research Grade	no HR	299.7	(81.7)	79.5	(10.7)	10.3	(16.1)	64.8	(39.1)
			HR	302.1	(81.2)	80.2	(10.2)	10.3	(16.0)	62.3	(37.3)
		Garmin	no HR	331.2	(60.3)	79.8	(10.7)	11.4	(17.3)	72.6	(40.8)
			HR	331.6	(59.9)	79.9	(10.4)	12.8	(17.8)	70.8	(39.8)
LSTM	Apple	Polysomnography		321.4	(81.1)	85.2	(11.0)	25.0	(29.1)	31.2	(29.3)
		Research Grade	no HR	311.0	(81.1)	82.3	(11.5)	26.6	(30.8)	40.1	(32.9)
			HR	312.7	(80.3)	82.6	(10.5)	26.8	(27.7)	38.2	(30.0)
		Apple	no HR	317.8	(77.6)	81.0	(13.1)	32.6	(36.0)	41.5	(38.3)
			HR	318.1	(80.9)	80.8	(13.7)	34.3	(38.2)	39.5	(34.1)
	Fitbit	Polysomnography		341.2	(82.3)	85.4	(12.6)	27.8	(30.0)	28.8	(25.1)
		Research grade	no HR	327.0	(83.6)	84.9	(10.6)	20.9	(21.6)	36.4	(28.8)
			HR	322.8	(83.7)	83.8	(11.2)	21.9	(22.1)	39.6	(30.2)
		Fitbit	no HR	333.9	(81.1)	83.6	(12.8)	27.4	(29.3)	36.5	(29.6)
			HR	325.4	(83.5)	81.3	(13.8)	29.4	(31.9)	43.0	(35.5)
	Garmin	Polysomnography		350.7	(65.7)	84.3	(10.7)	29.3	(29.9)	35.2	(28.0)
		Research grade	no HR	312.4	(82.2)	83.0	(10.8)	22.4	(19.7)	39.9	(32.3)
			HR	312.4	(82.7)	83.0	(10.7)	23.4	(22.8)	39.0	(29.4)
		Garmin	no HR	343.0	(64.5)	82.5	(10.6)	27.2	(25.7)	45.0	(38.2)
			HR	336.6	(72.6)	80.8	(13.2)	33.8	(43.5)	44.9	(34.5)

Algorithm	Dataset	Device
				Sleep stages													
TST	(SD)	SE		(SD)	SOL	(SD)	WASO	(SD)	Light	(SD)	Deep	(SD)	REM	(SD)
LR	Apple	Polysomnography		321.4	(81.1)	85.2	(11.0)	25.0	(29.1)	31.2	(29.3)	161.7	(52.1)	105.6	(46.0)	54.1	(24.3)
		Research Grade	no HR	312.7	(79.3)	82.6	(10.0)	10.1	(15.4)	54.8	(36.0)	207.7	(119.0)	103.3	(99.2)	1.7	(7.7)
			HR	317.6	(79.2)	83.9	(9.3)	9.7	(14.9)	50.4	(33.5)	151.6	(65.7)	132.2	(58.7)	33.8	(22.5)
		Apple	no HR	322.0	(74.1)	82.1	(10.7)	11.3	(20.3)	58.5	(39.2)	219.1	(102.7)	84.8	(89.3)	18.2	(21.2)
			HR	324.4	(73.9)	82.7	(10.3)	12.5	(21.0)	55.0	(36.5)	142.4	(46.9)	152.0	(50.3)	30.0	(23.3)
	Fitbit	Polysomnography		341.2	(82.3)	85.4	(12.6)	27.8	(30.0)	28.8	(25.1)	169.1	(50.4)	109.7	(36.3)	62.4	(28.1)
		Research grade	no HR	325.8	(80.3)	84.5	(8.6)	7.8	(13.5)	50.7	(30.0)	208.3	(107.3)	100.2	(94.7)	17.3	(30.1)
			HR	330.0	(80.0)	85.7	(8.1)	8.1	(13.4)	46.2	(28.3)	154.9	(55.3)	136.8	(53.8)	38.2	(30.1)
		Fitbit	no HR	337.5	(78.2)	84.5	(10.3)	8.6	(16.6)	51.8	(29.0)	255.6	(101.5)	78.5	(83.4)	3.4	(3.6)
			HR	339.3	(78.0)	85.0	(9.9)	9.8	(19.1)	48.7	(26.4)	163.4	(47.8)	139.3	(57.9)	36.6	(23.4)

	Garmin	Polysomnography		350.7	(65.7)	84.3	(10.7)	29.3	(29.9)	35.2	(28.0)	173.1	(47.0)	117.1	(41.1)	60.5	(27.0)
		Research Grade	no HR	309.0	(82.0)	82.0	(10.1)	8.6	(14.3)	57.1	(35.2)	189.1	(105.8)	112.2	(96.8)	7.8	(18.0)
			HR	315.0	(81.0)	83.7	(9.1)	8.3	(13.9)	51.5	(31.4)	134.0	(50.5)	135.3	(54.8)	45.7	(27.7)
		Garmin	no HR	334.5	(62.6)	80.6	(10.9)	8.4	(15.0)	72.3	(41.9)	214.2	(115.7)	108.5	(109.4)	11.8	(14.2)
HR			339.7	(61.5)	81.9	(10.2)	8.7	(15.0)	66.9	(39.4)	143.7	(48.1)	146.8	(56.3)	49.2	(25.5)
RF	Apple	Polysomnography		321.4	(81.1)	85.2	(11.0)	25.0	(29.1)	31.2	(29.3)	161.7	(52.1)	105.6	(46.0)	54.1	(24.3)
		Research Grade	no HR	301.7	(78.8)	79.8	(11.1)	10.2	(16.5)	65.8	(43.2)	75.6	(57.3)	145.6	(67.5)	80.5	(45.8)
			HR	308.4	(78.1)	81.6	(10.0)	10.5	(17.5)	58.7	(38.3)	106.6	(39.3)	95.4	(46.3)	106.4	(37.9)
		Apple	no HR	308.5	(74.7)	78.6	(12.4)	14.6	(22.7)	68.8	(47.4)	84.8	(44.4)	134.1	(54.8)	89.7	(47.4)
			HR	312.4	(73.4)	79.6	(11.6)	14.2	(22.0)	65.4	(43.6)	112.6	(36.6)	102.2	(42.9)	97.6	(40.6)
	Fitbit	Polysomnography		341.2	(82.3)	85.4	(12.6)	27.8	(30.0)	28.8	(25.1)	169.1	(50.4)	109.7	(36.3)	62.4	(28.1)
		Research grade	no HR	312.3	(79.1)	81.0	(9.5)	9.8	(15.5)	62.2	(33.8)	91.7	(64.8)	123.1	(67.1)	97.6	(49.9)
			HR	316.2	(78.9)	82.1	(9.0)	9.4	(15.4)	58.7	(32.1)	111.0	(38.8)	101.2	(47.0)	104.1	(43.9)
		Fitbit	no HR	319.7	(76.4)	80.0	(10.9)	14.6	(22.9)	63.5	(29.3)	93.4	(55.4)	125.3	(58.1)	101.0	(44.3)
			HR	323.3	(76.4)	80.9	(10.6)	14.2	(21.3)	60.3	(29.4)	114.7	(36.0)	102.5	(47.7)	106.2	(40.8)
	Garmin	Polysomnography		350.7	(65.7)	84.3	(10.7)	29.3	(29.9)	35.2	(28.0)	173.1	(47.0)	117.1	(41.1)	60.5	(27.0)
		Research Grade	no HR	305.3	(80.5)	81.0	(9.6)	8.9	(15.4)	60.5	(34.1)	114.0	(40.9)	94.6	(42.9)	96.7	(38.7)
			HR	298.9	(82.2)	79.3	(11.1)	9.3	(14.5)	66.6	(41.6)	66.9	(47.6)	140.6	(66.7)	91.4	(49.8)
		Garmin	no HR	329.3	(61.0)	79.4	(10.9)	11.6	(17.6)	74.3	(42.1)	77.8	(47.0)	161.2	(60.3)	90.2	(48.1)
			HR	333.8	(60.1)	80.4	(10.3)	11.3	(17.7)	70.2	(38.8)	111.7	(39.0)	112.9	(52.4)	109.1	(37.2)
LSTM	Apple	Polysomnography		321.4	(81.1)	85.2	(11.0)	25.0	(29.1)	31.2	(29.3)	161.7	(52.1)	105.6	(46.0)	54.1	(24.3)
		Research Grade	no HR	309.6	(82.8)	81.8	(12.3)	25.8	(28.5)	42.3	(36.4)	146.3	(51.8)	108.4	(40.5)	54.9	(27.7)
			HR	320.8	(82.2)	84.9	(11.5)	23.4	(25.8)	33.4	(38.6)	159.1	(51.7)	105.1	(42.5)	56.6	(24.1)
		Apple	no HR	317.7	(77.7)	80.9	(12.8)	33.2	(36.6)	40.9	(35.8)	150.7	(48.9)	114.3	(36.5)	52.7	(30.5)
			HR	327.8	(78.8)	83.1	(13.1)	30.3	(35.2)	33.7	(32.1)	161.0	(50.5)	113.8	(39.4)	53.0	(27.3)
	Fitbit	Polysomnography		341.2	(82.3)	85.4	(12.6)	27.8	(30.0)	28.8	(25.1)	169.1	(50.4)	109.7	(36.3)	62.4	(28.1)
		Research grade	no HR	325.7	(83.6)	84.4	(10.5)	21.3	(21.6)	37.3	(28.1)	147.0	(52.4)	111.1	(34.6)	67.5	(29.0)
			HR	334.4	(81.9)	86.8	(8.9)	22.0	(21.7)	27.9	(25.9)	161.7	(51.5)	105.3	(32.5)	67.3	(28.4)
		Fitbit	no HR	332.7	(81.3)	83.3	(12.9)	27.2	(29.5)	37.9	(31.1)	155.5	(49.0)	110.3	(33.9)	66.9	(28.8)
			HR	345.8	(82.5)	86.5	(10.6)	23.5	(26.2)	28.5	(31.8)	168.1	(54.2)	108.9	(34.3)	68.8	(29.5)

	Garmin	Polysomnography		350.7	(65.7)	84.3	(10.7)	29.3	(29.9)	35.2	(28.0)	173.1	(47.0)	117.1	(41.1)	60.5	(27.0)
		Research grade	no HR	312.4	(83.6)	82.9	(11.2)	22.8	(22.7)	39.5	(31.3)	139.2	(49.6)	113.5	(41.9)	59.7	(32.1)
			HR	320.7	(83.2)	85.2	(10.3)	22.6	(22.4)	31.4	(27.6)	152.0	(52.5)	109.3	(43.4)	59.5	(31.1)
		Garmin	no HR	341.6	(65.6)	82.0	(10.8)	28.9	(25.1)	44.7	(35.5)	160.8	(46.2)	116.9	(32.4)	63.9	(31.2)
HR			348.3	(65.4)	83.8	(10.5)	29.1	(25.5)	37.8	(34.8)	160.0	(51.9)	120.6	(39.0)	67.7	(31.7)

*Note*: “Research Grade Apple Dataset with HR” means the ActiGraph GT9X in conjunction with the PSG ECG channel, using the same pool of participants that also wore an Apple Watch, “Research Grade Fitbit Dataset with HR” means the ActiGraph GT9X in conjunction with the PSG ECG channel, using the same pool of participants that also wore an Fitbit, “Research Grade Garmin Dataset with HR” means the ActiGraph GT9X in conjunction with the PSG ECG channel, using the same pool of participants that also wore an Garmin.

Abbreviations: HR, heartrate; LR, Logistic Regression; LSTM, long short‐term memory; REM, rapid eye movement; RF, Random Forest; SD, standard deviation; SE, Sleep efficiency; SOL Sleep onset latency; TST, Total Sleep Time; WASO, Wake after sleep onset.

### 3.1 Evaluating LSTM Performance

**FIGURE 1:**
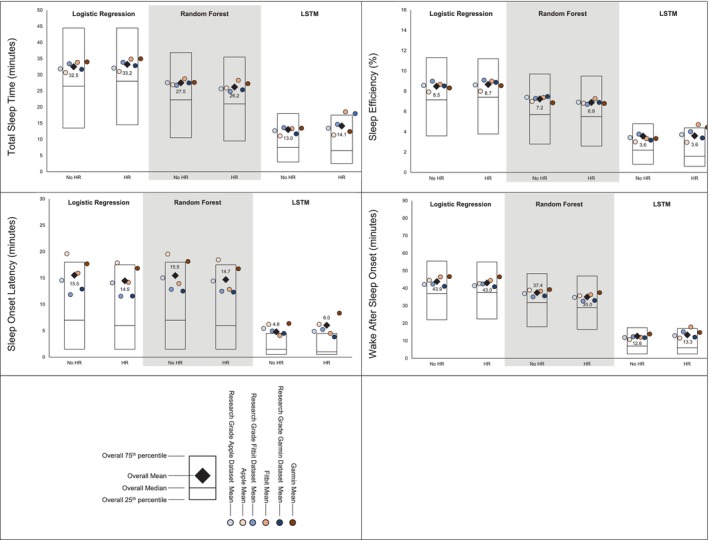
Mean absolute error for total sleep time, sleep efficiency, sleep onset latency, & wake after sleep onset by machine learning model and inclusion of heart rate when classifying sleep as binary. All values in minutes except for sleep effeciencey which is in percent, lower values represent better agreement with the criterion polysomnography (i.e., lower mean absolute error), “Research Grade Apple Dataset with HR” means the ActiGraph GT9X in conjunction with the PSG ECG channel, using the same pool of participants that also wore an Apple Watch, “Research Grade Fitbit Dataset with HR” means the ActiGraph GT9X in conjunction with the PSG ECG channel, using the same pool of participants that also wore an Fitbit, “Research Grade Garmin Dataset with HR” means the ActiGraph GT9X in conjunction with the PSG ECG channel, using the same pool of participants that also wore an Garmin. “HR” heartrate, “LSTM” long short‐term memory.

**FIGURE 2:**
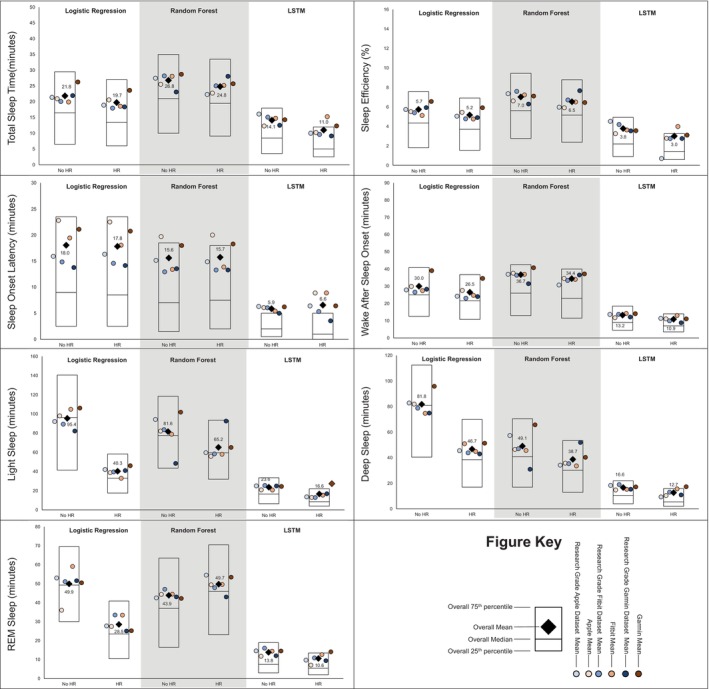
Mean absolute error for total sleep time, sleep efficiency, sleep onset latency, wake after sleep onset, light sleep time, deep sleep time, & REM sleep time by machine learning model and inclusion of heartrate when classifying sleep as sleep stage. All values in minutes except for sleep effeciencey which is in percent, lower values represent better agreement with the criterion polysomnography (i.e., lower mean absolute error), “Research Grade Apple Dataset with HR” means the ActiGraph GT9X in conjunction with the PSG ECG channel, using the same pool of participants that also wore an Apple Watch, “Research Grade Fitbit Dataset with HR” means the ActiGraph GT9X in conjunction with the PSG ECG channel, using the same pool of participants that also wore an Fitbit, “Research Grade Garmin Dataset with HR” means the ActiGraph GT9X in conjunction with the PSG ECG channel, using the same pool of participants that also wore an Garmin. “HR” heartrate, “LSTM” long short‐term memory, “REM” rapid eye movement.

**FIGURE 3:**
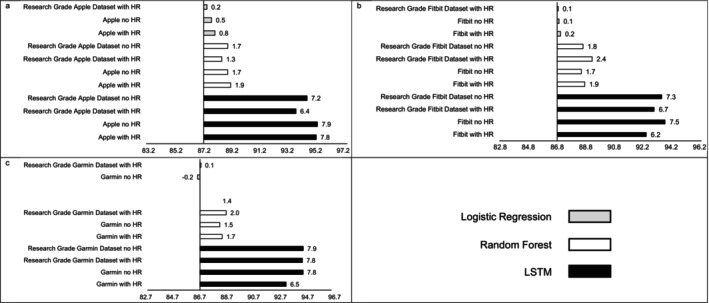
Accuracy for (a) Apple, (b) Fitbit, and (c) Garmin by machine learning model and inclusion of heartrate. Reference accuracy is always logistic regression with no heartrate, all values are percentages, “Research Grade Apple Dataset with HR” means the ActiGraph GT9X in conjunction with the PSG ECG channel, using the same pool of participants that also wore an Apple Watch, “Research Grade Fitbit Dataset with HR” means the ActiGraph GT9X in conjunction with the PSG ECG channel, using the same pool of participants that also wore an Fitbit, “Research Grade Garmin Dataset with HR” means the ActiGraph GT9X in conjunction with the PSG ECG channel, using the same pool of participants that also wore an Garmin. “HR” heartrate, “LSTM” long short‐term memory.

**FIGURE 4:**
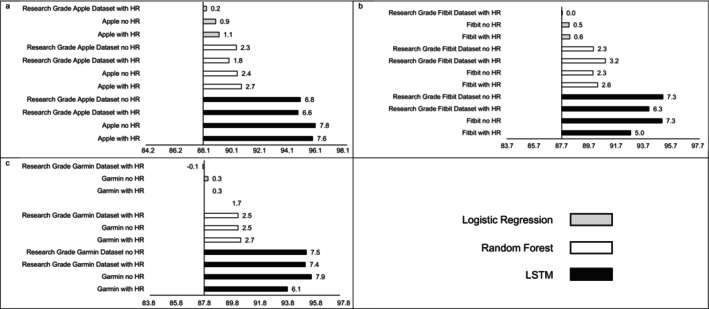
Sensitivity for (a) Apple, (b) Fitbit, and (c) Garmin by machine learning model and inclusion of heartrate. Reference sensitivity is always logistic regression with no heartrate, all values are percentages, “Research Grade Apple Dataset with HR” means the ActiGraph GT9X in conjunction with the PSG ECG channel, using the same pool of participants that also wore an Apple Watch, “Research Grade Fitbit Dataset with HR” means the ActiGraph GT9X in conjunction with the PSG ECG channel, using the same pool of participants that also wore an Fitbit, “Research Grade Garmin Dataset with HR” means the ActiGraph GT9X in conjunction with the PSG ECG channel, using the same pool of participants that also wore an Garmin. “HR” heartrate, “LSTM” long short‐term memory.

**FIGURE 5:**
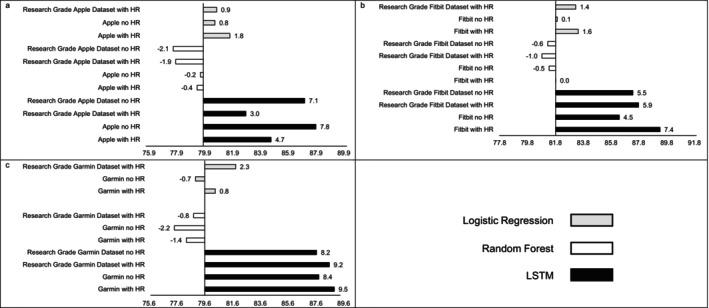
Specificity for (a) Apple, (b) Fitbit, and (c) Garmin by machine learning model and inclusion of heartrate. Reference specfificy is always logistic regression with no heartrate, all values are percentages, “Research Grade Apple Dataset with HR” means the ActiGraph GT9X in conjunction with the PSG ECG channel, using the same pool of participants that also wore an Apple Watch, “Research Grade Fitbit Dataset with HR” means the ActiGraph GT9X in conjunction with the PSG ECG channel, using the same pool of participants that also wore an Fitbit, “Research Grade Garmin Dataset with HR” means the ActiGraph GT9X in conjunction with the PSG ECG channel, using the same pool of participants that also wore an Garmin. Abbreviations: “HR” heartrate, “LSTM” long short‐term memory.

MAE estimates comparing LSTM performance to LR and RF performance are presented in Figure 1, while estimates for classifying sleep by sleep stage are presented in Figure 2. Across sleep metrics, LSTM produced the estimates with the lowest MAE, followed by RF, and then LR. For instance, LSTM total sleep time MAE ranged from 13.0 to 14.1, while for LR, total sleep time MAE ranged from 32.5 to 33.2, and for RF, total sleep time MAE ranged from 26.2 to 27.5 This pattern held across devices and predicted outcomes (i.e., sleep efficiency, sleep onset latency, wake after sleep onset, light sleep, deep sleep, REM sleep). Accuracy, sensitivity, and specificity for binary prediction of sleep and sleep stage are presented in Table 3. Figures 3, 4, 5 present the change in accuracy, sensitivity, and specificity when using different models for binary sleep/wake prediction. LSTM produced the highest accuracy, sensitivity, and specificity, followed by RF and LR. For instance, LSTM accuracy ranged from 6.2% to 7.9% points higher than LR, while RF ranged from 1.3% to 2.4% points higher than LR.

**TABLE 3:** Accuracy, sensitivity, and specificity by model and dataset.

			Binary (Sleep/Wake)													
			Acc	(SD)	Sen	(SD)	Spec	SD	Wake Acc	(SD)	Wake Sen	(SD)	Wake Spec	(SD)	Light Acc	(SD)
LR	Research Grade	no HR	87.2	(7.1)	88.1	(8.0)	81.8	(15.2)	89.4	(6.1)	73.3	(17.6)	92.1	(5.5)	55.5	(8.3)
	*Apple Dataset*	HR	87.4	(6.9)	88.4	(7.4)	82.7	(15.3)	90.1	(5.8)	71.5	(18.1)	93.4	(4.6)	63.9	(7.7)
	Research Grade	no HR	86.8	(7.2)	87.7	(7.9)	79.9	(16.6)	89.5	(5.6)	69.0	(20.2)	92.4	(4.9)	53.5	(6.8)
	*Fitbit Dataset*	HR	86.9	(7.1)	87.8	(7.5)	81.4	(16.5)	90.3	(5.2)	67.3	(20.9)	93.6	(3.8)	63.3	(7.5)
	Research Grade	no HR	86.7	(7.1)	87.8	(7.8)	79.6	(16.5)	89.0	(6.2)	72.5	(18.8)	91.5	(6.2)	55.8	(9.2)
	*Garmin Dataset*	HR	86.8	(7.3)	87.7	(7.9)	81.9	(15.3)	89.9	(5.2)	69.7	(20.0)	93.0	(4.4)	64.4	(7.6)
	Apple	no HR	87.7	(7.8)	89.0	(7.9)	82.6	(14.0)	89.5	(6.9)	71.4	(17.9)	93.2	(5.1)	54.4	(8.5)
		HR	87.9	(7.9)	89.2	(7.7)	83.6	(14.1)	89.8	(6.8)	70.5	(18.2)	93.9	(4.7)	64.3	(7.8)
	Fitbit	no HR	87.0	(6.4)	88.3	(6.9)	80.1	(15.2)	89.7	(4.8)	65.6	(20.5)	93.4	(3.8)	54.0	(8.9)
		HR	87.1	(6.4)	88.3	(6.9)	81.5	(14.7)	90.0	(5.0)	64.9	(21.0)	93.9	(3.4)	63.4	(8.7)
	Garmin	no HR	86.6	(8.3)	88.0	(9.1)	78.9	(18.0)	87.7	(8.0)	72.8	(18.5)	90.4	(8.3)	55.2	(8.1)
		HR	86.7	(8.3)	88.1	(9.1)	80.4	(17.8)	88.4	(7.6)	71.1	(19.0)	91.6	(7.4)	64.6	(6.6)
RF	Research Grade	no HR	88.4	(7.1)	89.9	(7.7)	79.9	(16.2)	88.3	(6.8)	80.1	(16.3)	89.8	(7.5)	58.8	(7.7)
	*Apple Dataset*	HR	88.8	(6.7)	90.4	(6.9)	79.7	(16.8)	89.5	(6.1)	77.8	(16.9)	91.6	(5.8)	63.7	(7.6)
	Research Grade	no HR	88.6	(6.3)	90.0	(6.7)	79.4	(16.7)	88.3	(6.3)	79.3	(16.7)	89.7	(6.6)	57.1	(7.9)
	*Fitbit Dataset*	HR	89.3	(6.1)	90.9	(5.8)	79.0	(17.6)	88.9	(6.2)	77.6	(17.4)	90.7	(6.0)	63.0	(7.5)
	Research Grade	no HR	88.1	(6.7)	89.5	(7.5)	79.0	(16.6)	89.0	(6.0)	77.0	(17.5)	90.9	(6.0)	65.5	(7.4)
	*Garmin Dataset*	HR	88.7	(6.4)	90.3	(6.8)	78.8	(17.2)	87.9	(7.3)	79.0	(16.4)	89.2	(8.2)	57.9	(8.9)
	Apple	no HR	88.8	(7.9)	90.5	(7.7)	81.6	(14.6)	89.1	(8.0)	80.9	(15.2)	90.9	(8.0)	61.5	(7.7)
		HR	89.0	(7.6)	90.8	(7.2)	81.3	(14.9)	89.7	(7.2)	79.6	(15.3)	91.8	(6.5)	64.2	(8.1)
	Fitbit	no HR	88.5	(5.7)	90.0	(5.9)	79.5	(16.4)	88.6	(5.8)	78.8	(16.2)	90.2	(6.0)	59.5	(8.4)
		HR	88.7	(5.9)	90.3	(6.1)	79.9	(16.3)	89.2	(5.4)	77.3	(17.1)	91.1	(5.3)	63.1	(9.0)
	Garmin	no HR	88.3	(7.2)	90.3	(7.6)	77.4	(18.8)	87.9	(7.4)	78.0	(18.4)	89.7	(8.1)	59.5	(7.8)
		HR	88.4	(7.1)	90.4	(7.4)	78.3	(18.9)	88.6	(6.9)	76.6	(18.9)	90.8	(7.1)	64.6	(7.1)
LSTM	Research Grade	no HR	94.4	(5.2)	94.9	(6.2)	88.8	(10.9)	92.8	(6.0)	85.0	(13.6)	93.5	(8.1)	79.8	(11.1)
	*Apple Dataset*	HR	93.6	(4.9)	94.7	(4.9)	84.7	(16.7)	93.6	(6.2)	73.8	(20.8)	95.9	(6.2)	87.3	(10.5)
	Research Grade	no HR	94.1	(5.7)	95.1	(6.7)	85.4	(17.2)	93.0	(5.1)	82.2	(16.1)	94.1	(6.6)	79.1	(11.5)
	*Fitbit Dataset*	HR	93.6	(5.1)	94.0	(7.2)	85.8	(14.7)	93.5	(6.2)	72.0	(22.6)	96.0	(4.1)	86.9	(10.5)
	Research Grade	no HR	94.6	(4.1)	95.3	(4.5)	87.9	(12.7)	93.8	(4.6)	85.1	(15.0)	94.7	(5.2)	82.7	(11.6)
	*Garmin Dataset*	HR	94.5	(4.6)	95.2	(4.8)	88.8	(12.2)	94.0	(6.0)	76.6	(21.5)	96.2	(4.6)	87.5	(10.4)
	Apple	no HR	95.1	(6.6)	95.9	(5.7)	89.6	(12.0)	94.5	(6.0)	86.7	(13.2)	95.6	(5.7)	84.6	(10.9)
		HR	95.0	(5.0)	95.7	(6.0)	87.2	(14.0)	94.1	(6.8)	76.6	(18.6)	96.6	(6.3)	88.7	(9.5)
	Fitbit	no HR	94.3	(4.6)	95.0	(7.7)	84.5	(15.9)	93.5	(4.5)	80.8	(16.5)	94.5	(5.9)	81.7	(10.5)
		HR	93.0	(6.3)	92.7	(11.3)	87.3	(13.1)	92.7	(7.9)	66.8	(23.6)	96.1	(5.8)	86.1	(11.1)
	Garmin	no HR	94.6	(4.7)	95.6	(4.4)	88.0	(13.7)	93.6	(5.1)	85.5	(13.3)	94.7	(5.1)	81.9	(9.8)
		HR	93.3	(9.2)	93.9	(10.3)	89.2	(11.2)	93.9	(5.2)	79.2	(19.5)	95.9	(4.4)	84.1	(10.9)

			Sleep Stage (Wake, Light, Deep, REM)															
			Light Sen	(SD)	Light Spec	(SD)	Deep Acc	(SD)	Deep Sen	(SD)	Deep Spec	(SD)	REM Acc	(SD)	REM Sen	(SD)	REM Spec	(SD)
LR	Research Grade	no HR	59.4	(28.7)	50.1	(27.3)	65.6	(12.1)	38.2	(36.0)	75.1	(23.7)	85.1	(6.5)	1.0	(4.3)	99.7	(1.5)
	*Apple Dataset*	HR	53.7	(17.3)	70.7	(14.0)	72.9	(8.7)	64.1	(21.2)	75.9	(12.3)	82.3	(6.8)	20.8	(21.1)	93.0	(4.7)
	Research Grade	no HR	58.1	(26.0)	48.5	(24.4)	66.2	(10.6)	36.0	(32.0)	78.1	(20.3)	81.6	(7.8)	7.3	(12.9)	96.1	(7.0)
	*Fitbit Dataset*	HR	54.0	(15.0)	69.6	(13.6)	73.2	(8.6)	64.9	(19.3)	76.6	(11.1)	81.1	(6.8)	22.2	(21.5)	92.4	(5.7)
	Research Grade	no HR	56.4	(25.4)	53.8	(25.4)	66.2	(10.8)	41.7	(34.3)	75.3	(20.6)	83.9	(7.0)	3.6	(8.9)	98.3	(3.8)
	*Garmin Dataset*	HR	50.3	(14.9)	74.1	(11.6)	73.8	(8.6)	66.0	(18.1)	76.8	(12.1)	81.8	(6.7)	28.3	(24.6)	91.2	(5.3)
	Apple	no HR	63.4	(26.6)	47.6	(24.6)	67.2	(11.9)	30.8	(32.1)	82.6	(19.0)	85.5	(6.6)	8.7	(10.0)	96.2	(4.7)
		HR	50.7	(14.8)	73.2	(11.9)	72.6	(8.3)	69.1	(18.9)	74.4	(10.7)	84.9	(6.8)	14.2	(20.2)	94.1	(4.8)

	Fitbit	no HR	69.8	(22.6)	39.9	(23.7)	68.4	(10.9)	28.0	(31.0)	83.4	(18.1)	84.2	(6.4)	1.4	(2.0)	99.2	(1.0)
		HR	55.2	(13.5)	69.0	(12.0)	72.7	(8.5)	62.4	(21.7)	76.7	(10.5)	81.5	(6.5)	20.8	(21.1)	92.6	(5.2)
	Garmin	no HR	58.5	(29.2)	51.8	(27.3)	66.5	(10.5)	35.3	(36.1)	78.6	(22.4)	84.3	(6.3)	4.8	(4.7)	97.5	(4.0)
HR		48.4	(15.2)	75.9	(10.6)	74.3	(9.2)	68.1	(19.8)	77.4	(14.3)	82.7	(6.3)	30.1	(24.8)	91.2	(5.4)
RF	Research Grade	no HR	24.6	(16.7)	83.6	(13.4)	67.4	(9.9)	59.2	(25.4)	70.1	(14.1)	73.1	(8.5)	30.5	(19.5)	80.2	(10.9)
	*Apple Dataset*	HR	40.4	(12.2)	80.9	(9.0)	77.2	(7.3)	53.5	(20.5)	86.2	(7.7)	74.5	(8.4)	60.4	(22.5)	77.0	(8.9)
	Research Grade	no HR	26.6	(16.6)	78.1	(17.3)	70.1	(8.5)	52.0	(26.7)	76.6	(12.2)	70.9	(8.9)	38.5	(20.0)	77.1	(11.6)
	*Fitbit Dataset*	HR	40.5	(11.8)	79.7	(10.4)	76.6	(8.0)	54.7	(20.0)	85.5	(9.4)	74.4	(8.6)	54.4	(24.0)	78.1	(10.2)
	Research Grade	no HR	44.6	(12.6)	79.9	(8.8)	78.5	(7.6)	55.5	(19.4)	87.5	(8.1)	77.3	(7.7)	57.5	(24.7)	80.4	(8.5)
	*Garmin Dataset*	HR	20.4	(12.8)	83.7	(12.9)	68.4	(9.0)	58.3	(22.9)	72.3	(13.3)	71.7	(8.6)	36.0	(20.7)	77.7	(11.5)
	Apple	no HR	28.9	(13.6)	83.2	(10.7)	70.9	(8.8)	58.8	(21.8)	76.0	(11.5)	76.1	(8.8)	42.3	(20.2)	80.5	(10.3)
		HR	41.3	(12.2)	79.7	(8.9)	76.7	(8.7)	55.2	(21.1)	86.0	(8.2)	77.1	(8.0)	56.1	(23.0)	79.9	(8.6)
	Fitbit	no HR	28.6	(14.0)	80.8	(13.4)	71.5	(8.4)	54.7	(22.2)	77.8	(11.7)	72.6	(7.9)	42.4	(17.2)	77.8	(10.1)
		HR	40.6	(12.1)	79.7	(10.1)	77.0	(8.0)	53.7	(21.5)	85.9	(8.4)	74.8	(7.6)	55.3	(23.3)	78.3	(9.0)
	Garmin	no HR	23.6	(12.5)	84.8	(11.4)	68.8	(9.7)	64.3	(23.6)	71.0	(15.0)	74.4	(8.7)	34.6	(18.5)	80.7	(10.6)
		HR	39.3	(13.4)	82.5	(8.8)	76.8	(8.7)	57.3	(21.4)	84.9	(11.6)	76.1	(9.7)	59.3	(24.0)	78.9	(10.6)
LSTM	Research Grade	no HR	70.6	(18.3)	86.2	(9.3)	89.4	(7.8)	81.8	(18.9)	91.8	(7.8)	89.7	(8.5)	66.5	(29.5)	94.1	(5.9)
	*Apple Dataset*	HR	83.7	(15.4)	89.9	(8.8)	94.5	(5.9)	89.4	(14.2)	96.1	(5.1)	94.2	(6.3)	82.6	(24.1)	96.2	(4.3)
	Research Grade	no HR	68.6	(19.5)	86.0	(10.9)	89.4	(7.9)	82.2	(17.5)	92.1	(7.7)	88.8	(8.4)	69.2	(27.1)	92.5	(6.5)

	*Fitbit Dataset*	HR	82.9	(15.0)	89.6	(9.2)	93.6	(7.3)	88.2	(14.6)	96.0	(6.0)	93.7	(6.6)	84.9	(20.4)	95.6	(5.8)
	Research Grade	no HR	73.3	(19.5)	89.2	(8.8)	91.5	(7.0)	88.0	(14.6)	92.7	(7.2)	92.2	(7.8)	75.3	(24.8)	95.1	(6.0)
	*Garmin Dataset*	HR	83.1	(15.3)	90.6	(9.4)	94.3	(6.1)	90.6	(13.5)	95.6	(5.9)	94.4	(6.5)	82.5	(24.0)	96.2	(5.5)
	Apple	no HR	77.7	(16.4)	89.3	(9.7)	92.1	(7.1)	87.0	(16.4)	94.1	(6.1)	93.2	(6.9)	74.2	(27.8)	95.7	(5.6)
		HR	84.9	(15.3)	90.7	(8.2)	94.2	(6.9)	90.2	(14.9)	95.8	(5.5)	95.8	(5.4)	85.1	(22.2)	97.2	(3.9)
	Fitbit	no HR	73.1	(16.9)	87.2	(8.9)	91.8	(6.4)	85.5	(14.2)	94.2	(5.6)	90.7	(7.6)	72.8	(25.4)	93.8	(5.8)
		HR	82.0	(16.1)	88.1	(10.6)	92.7	(7.9)	86.3	(16.5)	95.2	(7.2)	93.4	(7.3)	83.9	(21.6)	95.2	(5.7)
	Garmin	no HR	73.7	(17.3)	87.3	(8.7)	90.6	(8.3)	85.3	(13.4)	93.3	(7.7)	91.5	(7.0)	72.5	(26.3)	94.5	(5.5)
HR		76.7	(18.1)	89.4	(10.2)	92.2	(7.6)	89.2	(12.9)	93.7	(8.5)	93.0	(6.5)	81.3	(23.2)	94.9	(5.6)

*Note*: “Research Grade Apple Dataset with HR” means the ActiGraph GT9X in conjunction with the PSG ECG channel, using the same pool of participants that also wore an Apple Watch, “Research Grade Fitbit Dataset with HR” means the ActiGraph GT9X in conjunction with the PSG ECG channel, using the same pool of participants that also wore an Fitbit, “Research Grade Garmin Dataset with HR” means the ActiGraph GT9X in conjunction with the PSG ECG channel, using the same pool of participants that also wore an Garmin.

Abbreviations: Acc, accuracy; HR, heartrate; LR, Logistic Regression; LSTM, long short‐term memory; REM, rapid eye movement; RF, Random Forest; SD, standard deviation; Sen, sensitivity; Spec, specificity.

### 3.2 Evaluating the Impact of HR on Model Performance

Figures 1 and 2 also show the MAE of each model when not including HR and including HR for binary prediction of sleep and sleep stage prediction, respectively. Adding HR as a feature to prediction models did not substantially improve the binary prediction of sleep versus wake. For instance, when predicting binary sleep/wake using LR, sleep efficiency MAE was 8.5 without HR and 8.7 with HR. This pattern was consistent across sleep outcomes (i.e., sleep efficiency, sleep onset latency, and wake after sleep onset), devices (i.e., research grade, Apple, Fitbit, and Garmin), and models (i.e., LR, RF, and LSTM). However, especially for LR, HR improved the prediction of sleep stages. For instance, when predicting light sleep using LR without HR, MAE was 95.4. When HR was included, MAE was 40.3. This improvement was consistent for deep and REM sleep. While the improvement was the largest for LR, the pattern was also evident for RF and LSTM.

Similar to MAE, the addition of HR as a feature did not consistently improve accuracy (see Figure 3), sensitivity (see Figure 4), or specificity (see Figure 5) when predicting binary sleep. However, when predicting the sleep stage, accuracy, sensitivity, and specificity consistently improved across algorithms and devices. For instance, for LR with Apple, the inclusion of HR increased accuracy by 0.8 percentage points when predicting the sleep stage.

Bland–Altman plots for LSTM, the best performing model, are presented in Supplementary Figures 1–4.

## 4 Discussion

The aim of this paper was to explore the ability of LSTM to predict sleep and sleep stages from actigraphy alone and actigraphy combined with HR among children. Results showed that LSTM outperformed both LR and RF in all prediction scenarios. We also found that the addition of HR did not improve binary sleep prediction. However, including HR did improve performance when predicting the sleep stage.

An important finding of the current study is that LSTM outperformed LR and RF. Further, data in the current study suggest that LSTM substantially outperforms traditional count‐based approaches to predict sleep from actigraphy data. For instance, the (Sadeh et al. 1994) and (Cole et al. 1992) algorithms consistently demonstrate accuracies up to the mid‐80s (van Kooten et al. 2021) with specificity in the 60s (Goldstone et al. 2018). Our previous work applying Sadeh in this population demonstrated accuracies maxing out in the low‐to‐mid 80s with specificities in the mid‐70s (Weaver et al. 2023). In the current study, accuracy for LSTM was in the mid‐90s, and specificity was consistently in the mid‐to‐high 80s. Past studies have demonstrated specificity ranging from 37.0 to 70.7 (Fedorin et al. 2019). This demonstrates that LSTM is a promising approach not only for maximising accuracy but also specificity, one of the most significant weaknesses of actigraphy‐predicted sleep.

This study also demonstrated that LSTM shows promise for predicting sleep stage from actigraphy data alone. Predicting sleep stage from wrist actigraphy has been criticised as an approach because it relies on movement alone and does not include a physiological signal (Depner et al. 2020). However, past approaches predicting sleep stage from wrist actigraphy have not incorporated LSTM (Yuan et al. 2024). LSTM is uniquely positioned to predict sleep stage from wrist actigraphy because of its reliance on dependencies in the data over time (i.e., the current prediction depends on what occurred previously and what follows). For this reason, LSTM is ideal for the prediction of sleep stage, because it is phasic (i.e., composed of different phases) and ordered (i.e., N1 comes before N2 and N3 comes before REM). This strength is highlighted by the findings of the current study where LSTM predicted REM with accuracy ranging from 74.4 to 93.2 when using LSTM with actigraphy data alone. Past studies have been unable to predict sleep stage with actigraphy data with this same degree of accuracy. At least one study in adults using a variety of approaches (none of which were LSTM) to predict REM sleep from actigraphy data produced accuracy that ranged from 0.68 to 0.71 (Walch et al. 2019). Further, in the current study, the performance of the LSTM models with actigraphy alone produced better or equivalent accuracy estimates than previous approaches that incorporated both actigraphy and HR (i.e., accuracies of 0.77 (Fedorin et al. 2019), 0.69 (Beattie et al. 2017), 0.59 (Fonseca et al. 2017), 0.71 (Fonseca et al. 2018)) and is comparable to past studies that have used LSTM in adults to predict binary sleep (Sano et al. 2018). While these studies were with adults, the difference is striking, with the worst performing LSTM model outperforming the best models of different algorithms from past research.

Another key finding of this study is that the addition of HR as a feature did not consistently improve binary sleep/wake prediction. However, when predicting the sleep stage, HR improved model performance. For instance, when predicting light sleep via LR, MAE dropped from 95.4 min when not including HR to 40.3 min when HR was added. This pattern was consistent across different algorithms and agreement metrics. This finding is in line with previous studies that have successfully used actigraphy and HR metrics to predict sleep stage in adults (Fedorin et al. 2019; Walch et al. 2019; Beattie et al. 2017).

This is also one of the first LSTM studies in children. Further, this is one of the first studies to use this approach with data commonly available from wearables (i.e., actigraphy and HR) to predict sleep. Data from wearables is substantially easier to collect than PSG data (i.e., electroencephalogram, electromyography, and electrooculogram data) because it does not require high‐end equipment and highly trained sleep technicians to collect. Further, wearable data can be collected from participants in a more naturalistic setting that more closely resembles a typical night of sleep (i.e., no leads connected to the body, and wearable can be collected easily at home).

The current study has several strengths, which include the use of a gold standard criterion (i.e., PSG), including a large diverse sample of children, and the use of several machine learning models and data from both research grade and consumer wearable devices. These strengths enhance the generalisability and the confidence in the findings from the current study. Further, this study employed a device‐agnostic approach that relied on the underlying data collected from these devices rather than proprietary algorithms that are built in. However, the current study also has several limitations that should be considered when interpreting the results. For instance, the study was conducted in a lab setting over one night. Lab settings may reduce the ecological validity of the findings. Further, because of the lab setting, time in and out of bed was relatively constant, leading to relatively constant sleep onset and offset. Thus, the promising findings of LSTM to predict sleep stage may be contingent on these constant sleep onset and offset times, with sleep architecture (i.e., sleep staging) remaining relatively constant across participants. Thus, future studies should explore the performance of LSTM for prediction of sleep stage using actigraphy alone and actigraphy combined with HR when sleep onset and offset times vary and in special populations like shift workers or individuals with narcolepsy. The participants in the current study were also referred to the sleep lab by their paediatrician because of disrupted sleep. Thus, findings may not be generalisable to all children. Further, while a device‐agnostic approach was adopted, differences in hardware and software between different devices may influence results. Thus, studies that employ devices that were not included in the current study should verify the findings herein with those devices. This study was also limited by the inclusion, as HR in beats per minute alone was included as a feature, as this was the only feature available from the consumer wearable devices. Studies have shown that HR variability is important for predicting sleep stage (Lujan et al. 2021; Aktaruzzaman et al. 2017) and it may also be important for identifying binary sleep‐wake. Thus, including HR variability in future studies may further improve model performance.

The findings from this study indicate that LSTM shows considerable promise for improving the accuracy and specificity of binary sleep– wake prediction using actigraphy and HR data. Further, HR improved sleep stage prediction. LSTM was superior for predicting sleep stages compared to LR and RF and performed well even without including HR as a feature in this sample of participants. Future research should explore the performance of LSTM with accelerometry and HR in children without suspected sleep disruptions and in ambulatory settings.

## Author Contributions

**R. Glenn Weaver:** conceptualization, investigation, funding acquisition, writing – original draft, methodology, validation, visualization, writing – review and editing, supervision, resources. **James W. White:** supervision, writing – review and editing, project administration. **Olivia Finnegan:** writing – review and editing, supervision, project administration. **Hongpeng Yang:** formal analysis, software, data curation, writing – review and editing, writing – original draft. **Zifei Zhong:** formal analysis, data curation, writing – review and editing. **Keagan Kiely:** project administration, data curation, writing – review and editing. **Catherine Jones:** writing – review and editing, project administration, supervision. **Yan Tong:** writing – review and editing. **Srihari Nelakuditi:** writing – review and editing, supervision, project administration, formal analysis, resources, funding acquisition. **Rahul Ghosal:** writing – review and editing, formal analysis, supervision, resources. **David E. Brown:** writing – review and editing, funding acquisition. **Russ Pate:** funding acquisition, writing – review and editing. **Gregory J. Welk:** funding acquisition, writing – review and editing. **Massimiliano de Zambotti:** funding acquisition, writing – review and editing. **Yuan Wang:** writing – review and editing, funding acquisition. **Sarah Burkart:** writing – review and editing, data curation, project administration, supervision. **Elizabeth L. Adams:** writing – review and editing, data curation, supervision, formal analysis. **Bridget Armstrong:** writing – review and editing, formal analysis, data curation, supervision. **Michael W. Beets:** visualization, writing – review and editing, formal analysis, supervision.

## Conflicts of Interest

The authors declare no conflicts of interest.

## Supporting information


**Data S1** Supporting Information.

## Data Availability

The data that support the findings of this study are available on request from the corresponding author. The data are not publicly available due to privacy or ethical restrictions.
