# Supplementary material for: Predicting Sleep and Sleep Stage in Children Using Actigraphy and Heartrate via a Long Short-Term Memory Deep Learning Algorithm: A Performance Evaluation
Source: J Sleep Res. Author manuscript; Available in PMC 2026 Jan 6. (PMC12766639; doi:10.1111/jsr.70149)
Supplement: Supplementary file 1 [file NIHMS2127699-supplement-Supplementary_material.docx]

| Supplementary Table 1. Features included in the machine learning models |  |  |  |
| --- | --- | --- | --- |
| Feature | Definition | Model/s in which Feature was Included | Reference |
| enmoTrunc | Euclidean norm minus one truncated to zero | RF/LR | [1] |
| enmoAbs | Absolute value of Euclidean norm | RF/LR | [1] |
| xMean | Mean of x axis | RF/LR | [1] |
| yMean | Mean of y axis | RF/LR | [1] |
| zMean | Mean of z axis | RF/LR | [1] |
| xRange | Range of x axis | RF/LR | [1] |
| yRange | Range of y axis | RF/LR | [1] |
| zRange | Range of z axis | RF/LR | [1] |
| xStd | Standard deviation of x axis | RF/LR | [1] |
| yStd | Standard deviation of y axis | RF/LR | [1] |
| zStd | Standard deviation of z axis | RF/LR | [1] |
| xyCov | Covariance of x and y | RF/LR | [1] |
| xzCov | Covariance of x and z | RF/LR | [1] |
| yzCov | Covariance of y and z | RF/LR | [1] |
| entropy | spectral entropy of Vector Magnitude | RF/LR | [1] |
| MPD | Mean power deviation of Vector Magnitude | RF/LR | [1] |
| skew | Skewness of Vector Magnitude | RF/LR | [1] |
| kurt | Kurtosis of Vector Magnitude | RF/LR | [1] |
| avgArmAngel | Average of ARM angel | RF/LR | [1] |
| avgArmAngelAbsDiff | Average of Absolute difference of ARM angel | RF/LR | [1] |
| f1 | Dominant frequency | RF/LR | [1] |
| p1 | Power of the dominant frequency f1 | RF/LR | [1] |
| f2 | Second dominant frequency | RF/LR | [1] |
| p2 | Power of f2 | RF/LR | [1] |
| f625 | Dominant frequency between 0.6 -2.5 Hz | RF/LR | [1] |
| p625 | The power corresponding with f625 | RF/LR | [1] |
| totalPower | The total power for the frequencies between 0.3 - 15 Hz | RF/LR | [1] |
| fMean | Mean of Vector Magnitude | RF/LR | [2] |
| fStd | Standard deviation of Vector Magnitude | RF/LR | [2] |
| fCoefVariation | Coefficient of variation of Vector Magnitude | RF/LR | [2] |
| fMedian | Median of Vector Magnitude | RF/LR | [2] |
| fMin | Minimum of Vector Magnitude | RF/LR | [2] |
| fMax | Maximum of Vector Magnitude | RF/LR | [2] |
| f25thP | 25th percentile of Vector Magnitude | RF/LR | [2] |
| f75thP | 75th percentile of Vector Magnitude | RF/LR | [2] |
| fAutocorr | 1-s lag autocorrelation of Vector Magnitude | RF/LR | [2] |
| fCorrxy | Correlation between x and y | RF/LR | [2] |
| fCorrxz | Correlation between x and z | RF/LR | [2] |
| fCorryz | Correlation between y and z | RF/LR | [2] |
| fAvgRoll | Average roll | RF/LR | [2] |
| fAvgPitch | Average pitch | RF/LR | [2] |
| fAvgYaw | Average yaw | RF/LR | [2] |
| fSdRoll | Standard deviation of roll | RF/LR | [2] |
| fSdPitch | Standard deviation of pitch | RF/LR | [2] |
| fSdYaw | Standard deviation of yaw | RF/LR | [2] |
| fRollG | Roll of gravity component | RF/LR | [2] |
| fPitchG | Pitch of gravity component | RF/LR | [2] |
| fYawG | Yaw of gravity component | RF/LR | [2] |
| fFmax | Dominant frequency | RF/LR | [2] |
| fPmax | Power at dominant frequency | RF/LR | [2] |
| fFmaxBand | Dominant frequency within band of 0.3 Hz and 3Hz | RF/LR | [2] |
| fPmaxBand | Power at dominant frequency within band of 0.3 Hz and 3Hz | RF/LR | [2] |
| fEntropy | Entropy of the frequency domain signal | RF/LR | [2] |
| vFFT0 | The power in each frequency 1 of Vector Magnitude | RF/LR/LSTM | [2] |
| vFFT1 | The power in each frequency 2 of Vector Magnitude | RF/LR/LSTM | [2] |
| vFFT2 | The power in each frequency 3 of Vector Magnitude | RF/LR/LSTM | [2] |
| vFFT3 | The power in each frequency 4 of Vector Magnitude | RF/LR/LSTM | [2] |
| vFFT4 | The power in each frequency 5 of Vector Magnitude | RF/LR/LSTM | [2] |
| vFFT5 | The power in each frequency 6 of Vector Magnitude | RF/LR/LSTM | [2] |
| vFFT6 | The power in each frequency 7 of Vector Magnitude | RF/LR/LSTM | [2] |
| vFFT7 | The power in each frequency 8 of Vector Magnitude | RF/LR/LSTM | [2] |
| vFFT8 | The power in each frequency 9 of Vector Magnitude | RF/LR/LSTM | [2] |
| vFFT9 | The power in each frequency 10 of Vector Magnitude | RF/LR/LSTM | [2] |
| vFFT10 | The power in each frequency 11 of Vector Magnitude | RF/LR/LSTM | [2] |
| vFFT11 | The power in each frequency 12 of Vector Magnitude | RF/LR/LSTM | [2] |
| vFFT12 | The power in each frequency 13 of Vector Magnitude | RF/LR/LSTM | [2] |
| vFFT13 | The power in each frequency 14 of Vector Magnitude | RF/LR/LSTM | [2] |
| vFFT14 | The power in each frequency 15 of Vector Magnitude | RF/LR/LSTM | [2] |
| vFFT15 | The power in each frequency 16 of Vector Magnitude | RF/LR/LSTM |  |
| vFFT16 | The power in each frequency 17 of Vector Magnitude | RF/LR/LSTM |  |
| vFFT17 | The power in each frequency 18 of Vector Magnitude | RF/LR/LSTM |  |
| vFFT18 | The power in each frequency 19 of Vector Magnitude | RF/LR/LSTM |  |
| vFFT19 | The power in each frequency 20 of Vector Magnitude | RF/LR/LSTM |  |
| vFFT20 | The power in each frequency 21 of Vector Magnitude | RF/LR/LSTM |  |
| vFFT21 | The power in each frequency 22 of Vector Magnitude | RF/LR/LSTM |  |
| vFFT22 | The power in each frequency 23 of Vector Magnitude | RF/LR/LSTM |  |
| vFFT23 | The power in each frequency 24 of Vector Magnitude | RF/LR/LSTM |  |
| vFFT24 | The power in each frequency 25 of Vector Magnitude | RF/LR/LSTM |  |
| vFFT25 | The power in each frequency 26 of Vector Magnitude | RF/LR/LSTM |  |
| vFFT26 | The power in each frequency 27 of Vector Magnitude | RF/LR/LSTM |  |
| vFFT27 | The power in each frequency 28 of Vector Magnitude | RF/LR/LSTM |  |
| vFFT28 | The power in each frequency 29 of Vector Magnitude | RF/LR/LSTM |  |
| vFFT29 | The power in each frequency 30 of Vector Magnitude | RF/LR/LSTM |  |
| BFEN | Bandpass-Filtered followed by Euclidean Norm | RF/LR | [3] |
| LFEN | Lowpass-Filtered followed by Euclidean Norm | RF/LR | [3] |
| LFENMO | Lowpass-Filtered followed by Euclidean Norm Minus One | RF/LR | [3] |
| HFEN | Euclidean norm of high-pass filtered signals | RF/LR | [3] |
| HFENplus | Euclidean norm of low-pass filtered signals minus gravity plus Euclidean norm of the high-pass filtered signals with resulting negative values rounded to zero | RF/LR | [3] |
| roll_med_acc_x | 5-second rolling median of a 10 Hertz version of x-axis | RF/LR | [3] |
| roll_med_acc_y | 5-second rolling median of a 10 Hertz version of y-axis | RF/LR | [3] |
| roll_med_acc_z | 5-second rolling median of a 10 Hertz version of z-axis | RF/LR | [3] |
| dev_roll_med_acc_x | Absolute difference between x-axis and 5-second rolling median of a 10 Hertz version of x-axis | RF/LR | [3] |
| dev_roll_med_acc_y | Absolute difference between y-axis and 5-second rolling median of a 10 Hertz version of y-axis | RF/LR | [3] |
| dev_roll_med_acc_z | Absolute difference between z-axis and 5-second rolling median of a 10 Hertz version of z-axis | RF/LR | [3] |
| angle_x | 5-second rolling median of a 10 Hertz version of the signals followed by atan(x / (sqrt(y^2^ + z^2^))) / (pi/180) | RF/LR | [3] |
| angle_y | 5-second rolling median of a 10 Hertz version of the signals followed by atan(y / (sqrt(x^2^ + z^2^))) / (pi/180) | RF/LR | [3] |
| angle_z | 5-second rolling median of a 10 Hertz version of the signals followed by atan(z/ (sqrt(x^2^ + y^2^))) / (pi/180) | RF/LR | [3] |
| ENMO | Euclidean norm minus one with resulting negative values rounded to zero | RF/LR | [3] |
| MAD | Absolute difference between Euclidean norm and epoch level average of the Euclidean norm | RF/LR | [3] |
| EN | Euclidean norm (vector magnitude) | RF/LR | [3] |
| ENMOa | Same as ENMO but with rectified negative values instead of rounded to zero | RF/LR | [3] |
| xFFT0 | The power in each frequency 1 of x axis | RF/LR/LSTM | [4] |
| xFFT1 | The power in each frequency 2 of x axis | RF/LR/LSTM | [4] |
| xFFT2 | The power in each frequency 3 of x axis | RF/LR/LSTM | [4] |
| xFFT3 | The power in each frequency 4 of x axis | RF/LR/LSTM | [4] |
| xFFT4 | The power in each frequency 5 of x axis | RF/LR/LSTM | [4] |
| xFFT5 | The power in each frequency 6 of x axis | RF/LR/LSTM | [4] |
| xFFT6 | The power in each frequency 7 of x axis | RF/LR/LSTM | [4] |
| xFFT7 | The power in each frequency 8 of x axis | RF/LR/LSTM | [4] |
| xFFT8 | The power in each frequency 9 of x axis | RF/LR/LSTM | [4] |
| xFFT9 | The power in each frequency 10 of x axis | RF/LR/LSTM | [4] |
| xFFT10 | The power in each frequency 11 of x axis | RF/LR/LSTM | [4] |
| xFFT11 | The power in each frequency 12 of x axis | RF/LR/LSTM | [4] |
| xFFT12 | The power in each frequency 13 of x axis | RF/LR/LSTM | [4] |
| xFFT13 | The power in each frequency 14 of x axis | RF/LR/LSTM | [4] |
| xFFT14 | The power in each frequency 15 of x axis | RF/LR/LSTM | [4] |
| xFFT15 | The power in each frequency 16 of x axis | RF/LR | [4] |
| xFFT16 | The power in each frequency 17 of x axis | RF/LR | [4] |
| xFFT17 | The power in each frequency 18 of x axis | RF/LR | [4] |
| xFFT18 | The power in each frequency 19 of x axis | RF/LR | [4] |
| xFFT19 | The power in each frequency 20 of x axis | RF/LR | [4] |
| xFFT20 | The power in each frequency 21 of x axis | RF/LR | [4] |
| xFFT21 | The power in each frequency 22 of x axis | RF/LR | [4] |
| xFFT22 | The power in each frequency 23 of x axis | RF/LR | [4] |
| xFFT23 | The power in each frequency 24 of x axis | RF/LR | [4] |
| xFFT24 | The power in each frequency 25 of x axis | RF/LR | [4] |
| xFFT25 | The power in each frequency 26 of x axis | RF/LR | [4] |
| xFFT26 | The power in each frequency 27 of x axis | RF/LR | [4] |
| xFFT27 | The power in each frequency 28 of x axis | RF/LR | [4] |
| xFFT28 | The power in each frequency 29 of x axis | RF/LR | [4] |
| xFFT29 | The power in each frequency 30 of x axis | RF/LR | [4] |
| yFFT0 | The power in each frequency 1 of y axis | RF/LR/LSTM | [4] |
| yFFT1 | The power in each frequency 2 of y axis | RF/LR/LSTM | [4] |
| yFFT2 | The power in each frequency 3 of y axis | RF/LR/LSTM | [4] |
| yFFT3 | The power in each frequency 4 of y axis | RF/LR/LSTM | [4] |
| yFFT4 | The power in each frequency 5 of y axis | RF/LR/LSTM | [4] |
| yFFT5 | The power in each frequency 6 of y axis | RF/LR/LSTM | [4] |
| yFFT6 | The power in each frequency 7 of y axis | RF/LR/LSTM | [4] |
| yFFT7 | The power in each frequency 8 of y axis | RF/LR/LSTM | [4] |
| yFFT8 | The power in each frequency 9 of y axis | RF/LR/LSTM | [4] |
| yFFT9 | The power in each frequency 10 of y axis | RF/LR/LSTM | [4] |
| yFFT10 | The power in each frequency 11 of y axis | RF/LR/LSTM | [4] |
| yFFT11 | The power in each frequency 12 of y axis | RF/LR/LSTM | [4] |
| yFFT12 | The power in each frequency 13 of y axis | RF/LR/LSTM | [4] |
| yFFT13 | The power in each frequency 14 of y axis | RF/LR/LSTM | [4] |
| yFFT14 | The power in each frequency 15 of y axis | RF/LR/LSTM | [4] |
| yFFT15 | The power in each frequency 16 of y axis | RF/LR | [4] |
| yFFT16 | The power in each frequency 17 of y axis | RF/LR | [4] |
| yFFT17 | The power in each frequency 18 of y axis | RF/LR | [4] |
| yFFT18 | The power in each frequency 19 of y axis | RF/LR | [4] |
| yFFT19 | The power in each frequency 20 of y axis | RF/LR | [4] |
| yFFT20 | The power in each frequency 21 of y axis | RF/LR | [4] |
| yFFT21 | The power in each frequency 22 of y axis | RF/LR | [4] |
| yFFT22 | The power in each frequency 23 of y axis | RF/LR | [4] |
| yFFT23 | The power in each frequency 24 of y axis | RF/LR | [4] |
| yFFT24 | The power in each frequency 25 of y axis | RF/LR | [4] |
| yFFT25 | The power in each frequency 26 of y axis | RF/LR | [4] |
| yFFT26 | The power in each frequency 27 of y axis | RF/LR | [4] |
| yFFT27 | The power in each frequency 28 of y axis | RF/LR | [4] |
| yFFT28 | The power in each frequency 29 of y axis | RF/LR | [4] |
| yFFT29 | The power in each frequency 30 of y axis | RF/LR | [4] |
| zFFT0 | The power in each frequency 1 of z axis | RF/LR/LSTM | [4] |
| zFFT1 | The power in each frequency 2 of z axis | RF/LR/LSTM | [4] |
| zFFT2 | The power in each frequency 3 of z axis | RF/LR/LSTM | [4] |
| zFFT3 | The power in each frequency 4 of z axis | RF/LR/LSTM | [4] |
| zFFT4 | The power in each frequency 5 of z axis | RF/LR/LSTM | [4] |
| zFFT5 | The power in each frequency 6 of z axis | RF/LR/LSTM | [4] |
| zFFT6 | The power in each frequency 7 of z axis | RF/LR/LSTM | [4] |
| zFFT7 | The power in each frequency 8 of z axis | RF/LR/LSTM | [4] |
| zFFT8 | The power in each frequency 9 of z axis | RF/LR/LSTM | [4] |
| zFFT9 | The power in each frequency 10 of z axis | RF/LR/LSTM | [4] |
| zFFT10 | The power in each frequency 11 of z axis | RF/LR/LSTM | [4] |
| zFFT11 | The power in each frequency 12 of z axis | RF/LR/LSTM | [4] |
| zFFT12 | The power in each frequency 13 of z axis | RF/LR/LSTM | [4] |
| zFFT13 | The power in each frequency 14 of z axis | RF/LR/LSTM | [4] |
| zFFT14 | The power in each frequency 15 of z axis | RF/LR/LSTM | [4] |
| zFFT15 | The power in each frequency 16 of x axis | RF/LR | [4] |
| zFFT16 | The power in each frequency 17 of z axis | RF/LR | [4] |
| zFFT17 | The power in each frequency 18 of z axis | RF/LR | [4] |
| zFFT18 | The power in each frequency 19 of z axis | RF/LR | [4] |
| zFFT19 | The power in each frequency 20 of z axis | RF/LR | [4] |
| zFFT20 | The power in each frequency 21 of z axis | RF/LR | [4] |
| zFFT21 | The power in each frequency 22 of z axis | RF/LR | [4] |
| zFFT22 | The power in each frequency 23 of z axis | RF/LR | [4] |
| zFFT23 | The power in each frequency 24 of z axis | RF/LR | [4] |
| zFFT24 | The power in each frequency 25 of z axis | RF/LR | [4] |
| zFFT25 | The power in each frequency 26 of z axis | RF/LR | [4] |
| zFFT26 | The power in each frequency 27 of z axis | RF/LR | [4] |
| zFFT27 | The power in each frequency 28 of z axis | RF/LR | [4] |
| zFFT28 | The power in each frequency 29 of z axis | RF/LR | [4] |
| zFFT29 | The power in each frequency 30 of z axis | RF/LR | [4] |
| xOffsetAngle | x-axis offset angle | LSTM | [5] |
| yOffsetAngle | y-axis offset angle | LSTM | [5] |
| zOffsetAngle | z-axis offset angle | LSTM | [5] |
| age | Age in years | LSTM | [5] |
| count | activity counts | LSTM | [6] |
| HR_std | Standard deviation of Heart Rate | LSTM | Created for this study |
| HR_min | Minimum of Heart Rate | LSTM | Created for this study |
| HR_max | Maximum of Heart Rate | LSTM | Created for this study |
| HR_ave | Average of Heart Rate | LSTM | Created for this study |
| HR_range | Range of Heart Rate | LSTM | Created for this study |
| Diagnoses_Mild | Mild Obstructive Sleep Apnea as Diagnosed by a Medical Doctor with a specialty in sleep and consistent with the American Academy of Sleep Medicine | LSTM | Created for this study |
| Diagnoses_Moderate | Moderate Obstructive Sleep Apnea as Diagnosed by a Medical Doctor with a specialty in sleep and consistent with the American Academy of Sleep Medicine | LSTM | Created for this study |
| Diagnoses_None | No Obstructive Sleep Apnea as Diagnosed by a Medical Doctor with a specialty in sleep and consistent with the American Academy of Sleep Medicine | LSTM | Created for this study |
| Diagnoses_Severe | Severe Obstructive Sleep Apnea as Diagnosed by a Medical Doctor with a specialty in sleep and consistent with the American Academy of Sleep Medicine | LSTM | Created for this study |
| *Abbreviations: “LR” Logistic Regression, “RF” Random Forest,  “LSTM”* *long short-term memory* |  |  |  |

| Supplementary Figure 1. Bland-Altman plots for LSTM predicting binary sleep by device and metric without heartrate |  |  |  |  |
| --- | --- | --- | --- | --- |
| Device | Total Sleep Time | Sleep Efficiency | Sleep Onset Latency | Wake After Sleep Onset |
| Research Grade *Apple Dataset* |  |  |  |  |
| Research Grade *Fitbit Dataset* |  |  |  |  |
| Research Grade *Garmin Dataset* |  |  |  |  |
| Apple |  |  |  |  |
| Fitbit |  |  |  |  |
| Garmin |  |  |  |  |

| Supplementary Figure 2. Bland-Altman plots for LSTM predicting binary sleep by device and metric with heartrate |  |  |  |  |
| --- | --- | --- | --- | --- |
| Device | Total Sleep Time | Sleep Efficiency | Sleep Onset Latency | Wake After Sleep Onset |
| Research Grade *Apple Dataset* |  |  |  |  |
| Research Grade *Fitbit Dataset* |  |  |  |  |
| Research Grade *Garmin Dataset* |  |  |  |  |
| Apple |  |  |  |  |
| Fitbit |  |  |  |  |
| Garmin |  |  |  |  |

| Supplementary Figure 3. Bland-Altman plots for LSTM predicting sleep stage by device and metric without heartrate |  |  |  |  |  |  |  |
| --- | --- | --- | --- | --- | --- | --- | --- |
| Device | Total Sleep Time | Sleep Efficiency | Sleep Onset Latency | Wake After Sleep Onset | Light Sleep | Deep Sleep | REM Sleep |
| Research Grade *Apple Dataset* |  |  |  |  |  |  |  |
| Research Grade *Fitbit Dataset* |  |  |  |  |  |  |  |
| Research Grade *Garmin Dataset* |  |  |  |  |  |  |  |
| Apple |  |  |  |  |  |  |  |
| Fitbit |  |  |  |  |  |  |  |
| Garmin |  |  |  |  |  |  |  |

| Supplementary Figure 4. Bland-Altman plots for LSTM predicting sleep stage by device and metric with heartrate |  |  |  |  |  |  |  |
| --- | --- | --- | --- | --- | --- | --- | --- |
| Device | Total Sleep Time | Sleep Efficiency | Sleep Onset Latency | Wake After Sleep Onset | Light Sleep | Deep Sleep | REM Sleep |
| Research Grade *Apple Dataset* |  |  |  |  |  |  |  |
| Research Grade *Fitbit Dataset* |  |  |  |  |  |  |  |
| Research Grade *Garmin Dataset* |  |  |  |  |  |  |  |
| Apple |  |  |  |  |  |  |  |
| Fitbit |  |  |  |  |  |  |  |
| Garmin |  |  |  |  |  |  |  |

1.	Doherty, A., D. Jackson, N. Hammerla, T. Plötz, P. Olivier, M.H. Granat, et al., *Large Scale Population Assessment of Physical Activity Using Wrist Worn Accelerometers: The UK Biobank Study.* PLoS One, 2017. **12**(2): p. e0169649.

2.	Ellis, K., J. Kerr, S. Godbole, J. Staudenmayer, and G. Lanckriet, *Hip and wrist accelerometer algorithms for free-living behavior classification.* Medicine and science in sports and exercise, 2016. **48**(5): p. 933.

3.	Migueles, J.H., A.V. Rowlands, F. Huber, S. Sabia, and V.T. van Hees, *GGIR: a research community–driven open source R package for generating physical activity and sleep outcomes from multi-day raw accelerometer data.* Journal for the Measurement of Physical Behaviour, 2019. **2**(3): p. 188-196.

4.	Willetts, M., S. Hollowell, L. Aslett, C. Holmes, and A. Doherty, *Statistical machine learning of sleep and physical activity phenotypes from sensor data in 96,220 UK Biobank participants.* Scientific reports, 2018. **8**(1): p. 7961.

5.	ActiGraph. *ActiGraph White Paper: Estimating Human Position with the ActiGraph GT3X Triaxial Activity Monitor*. 2022  [cited 2024 12/3]; 

6.	Walch, O., Y. Huang, D. Forger, and C. Goldstein, *Sleep stage prediction with raw acceleration and photoplethysmography heart rate data derived from a consumer wearable device.* Sleep, 2019. **42**(12): p. zsz180.
